# Controlled Synthesis of Tungsten Oxide Nanomaterials with Different Morphologies and Their Gas-Sensing Properties for Formaldehyde in Vegetables

**DOI:** 10.3390/bios15070400

**Published:** 2025-06-20

**Authors:** Weihao Wu, Yaochong Yang, Cheng Zhao, Xingyu Wang, Yitong Xie, Kexin Jiang, Huafeng Feng, Yongheng Zhu

**Affiliations:** 1 College of Food Science and Technology, Shanghai Ocean University, Shanghai 201306, China; 2 Henan Railway Food Safety Management Engineering Technology Research Center, Zhengzhou Railway Vocational & Technical College, Zhengzhou 451460, China; 3 Department of Food Science, Shanghai Business School, Shanghai 200235, China

**Keywords:** WO_3_, different morphologies, gas sensors, formaldehyde, vegetables, food safety

## Abstract

Formaldehyde is illegally applied to vegetables by vendors as a preservative to extend their shelf life, and it poses health risks to consumers. Herein, a series of WO_3_ with different morphologies were synthesized and employed as the sensing material in gas sensors to detect formaldehyde in vegetables rapidly. Among all the samples, the WO_3_ nanoplate sensor exhibited the best sensitivity (16.5@200 ppm), a rapid response/recovery time (10/12 s), superior selectivity, and a low limit of detection (500 ppb). This was mainly attributed to its abundant mesopores and large specific surface area, which enhanced the formaldehyde adsorption capacity and adsorption/desorption rates while providing more active sites, thereby improving the sensor’s response speed and resistance variation range. The WO_3_ nanoplate sensor also achieved reliable formaldehyde detection in actual vegetable samples (baby cabbage). This study provides systematic guidance for optimizing the gas-sensing performance of functional materials. It establishes a foundation for developing rapid, non-destructive formaldehyde detection technologies applicable for vegetable quality control.

## 1. Introduction

Formaldehyde, a toxic chemical widely present in the environment, has been classified as a Group 1 carcinogen by the International Agency for Research on Cancer [1]. However, due to its preservative, antibacterial, and bleaching properties, formaldehyde is often illegally added to food products, such as vegetables, to extend their shelf life or improve their appearance. Formaldehyde can react with various functional groups in proteins, disrupting their spatial structure and chemical bonds, which leads to protein denaturation [2]. Furthermore, formaldehyde can oxidize vitamins in vegetables, significantly reducing their nutritional value [3]. The ingestion of formaldehyde-contaminated vegetables can cause acute poisoning, manifesting as nausea, vomiting, abdominal pain, and diarrhea. In severe cases, it may lead to gastrointestinal bleeding, posing a significant threat to human health [4,5,6]. Currently, mainstream methods for detecting formaldehyde in food include fluorescence spectrophotometry [7], fluorometry [8], optical probes [9], spectroscopy [10], etc. However, these methods often suffer from drawbacks such as complex procedures, lengthy analysis times, and high costs, making them unsuitable for onsite, rapid detection [11]. Therefore, there is an urgent need to develop a fast and non-destructive method for detecting formaldehyde in food.

With the advancement of technology, new detection methods based on metal oxide semiconductor (MOS) gas sensors are gradually replacing traditional detection techniques due to their advantages of simple operation, low cost, high stability, and fast response/recovery times, making them a research hotspot in the field of formaldehyde detection [12,13,14]. The sensitive material, serving as a core component of a gas sensor, substantially impacts the sensor’s gas-sensitive characteristics [15,16,17]. As an important n-type semiconductor material, WO_3_ has widespread applications in gas sensing due to its unique chemical properties [18,19,20]. Zhao et al. successfully synthesized three-dimensional WO_3_ hollow microspheres assembled from single-crystal nanosheets. The fabricated gas sensor demonstrated a response value of 16.5 to 50 ppm triethylamine at 220 °C [21]. Additionally, Zhu et al. used mesoporous WO_3_ as the sensitive material for gas sensors to indirectly monitor *Listeria monocytogenes* by detecting 3-hydroxy-2-butanone [22]. It is widely recognized that the morphological structure of a material plays a crucial role in determining its gas-sensing performance. By adjusting the reaction conditions, the precise design of material dimensions (zero-dimensional quantum dots, one-dimensional nanowires, and two-dimensional nanosheets), pore structures (mesopores and macropores), and surface morphologies (core-shell structures and hierarchical flower-like structures) can be achieved [23,24,25]. Morphological control can not only alter the specific surface area of materials to provide more active sites for gas adsorption but also enhance gas-sensing performance by optimizing the surface adsorption energy and charge transport pathways [26]. For instance, the ZnO nanorods synthesized by Li et al. exhibited no response to 1 ppm NO_2_ [27]. In contrast, the flower-like ZnO and flake-like ZnO structures developed by Cai et al. and Lai et al. demonstrated the pronounced responses to 1 ppm and 0.5 ppm NO_2_, with response values of 54.8 and 76, respectively [28,29]. Therefore, modulating the morphology and structure of the sensitive material is an effective strategy for enhancing its gas-sensing performance.

This research synthesized a series of sensitive WO_3_ materials with diverse morphologies via a morphology-controlled strategy. Subsequently, microelectromechanical system (MEMS)-based gas sensors were fabricated using these materials for formaldehyde detection in vegetables. Quartz Crystal Microbalance (QCM) tests demonstrated that the WO_3_ nanoplate sensor exhibited the highest formaldehyde vapor adsorption capacity (0.83 wt%) and the fastest adsorption/desorption rates (0.083/0.068 wt%). Gas-sensing tests revealed that the optimal operating temperature for all materials in formaldehyde detection was 350 °C. WO_3_ nanoplates demonstrated the highest formaldehyde response (16.5@200 ppm) along with rapid response/recovery characteristics (10 s/12 s). Meanwhile, WO_3_ nanoplates demonstrated excellent repeatability and long-term stability. Additionally, various characterization methods were employed to examine the microstructure and surface chemistry of the gas-sensing materials, and their sensing mechanisms were investigated. This research offers new perspectives for enhancing the gas-sensing characteristics of sensitive materials, creating additional possibilities for the swift and effective monitoring of formaldehyde contamination in food and the environment.

## 2. Materials and Methods

### 2.1. Materials and Chemical Reagents

All analytical-grade chemicals and solvents were purchased and used without further purification. Pluronic P123 (PEG_20_-PPG_70_-PEG_20_, Mw = 5800, 99%), WCl_6_ (99.5%), and Na_2_WO_4_·2H_2_O (98%) were gained from Sigma-Aldrich, Saint Louis, MO, USA. Na_2_SO_4_ (99%), NaCl (99%), anhydrous ethanol (99.9%), and HCl (36%) were bought from Sinopharm Chemical Reagent Co., Ltd., Shanghai, China.

### 2.2. Synthesis of WO_3_ with Different Morphologies

A series of WO_3_ sensing materials with different morphologies were synthesized via a hydrothermal method by adding various additives. The typical synthesis procedure for WO_3_ nanoplates is described below. In total, 0.2 g of P123 was added to a mixed solution of 16 mL anhydrous ethanol and 450 μL deionized water under continuous stirring for 20 min. Subsequently, 0.4 g of WCl_6_ was added to the mixture, and stirring was continued for approximately 1 h to obtain a dark blue solution. The solution was then transferred into a 50 mL autoclave, sealed, and heated at 110 °C for 2 h. After the reaction, the precipitate was collected, washed, and centrifuged, followed by calcination at 400 °C for 2 h to obtain the WO_3_ nanoplates (the heating rate was 5 °C/min). The synthesis methods of WO_3_ with other morphologies are detailed in the Supporting Information.

### 2.3. Instruments

The crystal structure of the materials was characterized by X-ray diffraction (XRD) at 25 °C using a copper target (wavelength λ = 1.5418 Å). The microstructure was examined by scanning electron microscopy (SEM, Sigma 360, Carl Zeiss AG, Oberkochen, Germany) and high-resolution transmission electron microscopy (HRTEM; JEOL JEM-2011, JEOL Ltd., Tokyo, Japan). The solid-state UV-Vis diffuse reflectance spectra were obtained using a UV-Vis spectrophotometer equipped with an integrating sphere attachment (UV-vis 2550, Shimadzu Corporation, Kyoto, Japan). The chemical composition was analyzed using X-ray photoelectron spectroscopy (XPS; PHI-5000CESCA, ULVAC-PHI, Inc., Chiba, Japan). The Brunauer–Emmett–Teller (BET; ASAP2460, Micromeritics, Norcross, GA, USA) method determined the specific surface area with N_2_ as the adsorption–desorption medium.

### 2.4. Preparation of the Sensors

Microelectromechanical system (MEMS) gas sensors were fabricated following previously reported methodologies (Figures S1 and S2) [30,31]. The sensor device integrates a micro-heater module and an interdigitated electrode module. The micro-heater provides thermal activation for the sensing material, while the interdigitated electrode enables the sensitive detection of resistance variations during gas adsorption/desorption processes. The sensing material (10 mg) was thoroughly mixed with 1 mL ethanol in an agate mortar to form a homogeneous paste. This slurry was then uniformly coated onto the MEMS chip electrodes using a fine brush. Finally, the sensors were aged at 360 °C for 8 h to further enhance their stability.

## 3. Results

### 3.1. Material Characterization

**Figure 1:**
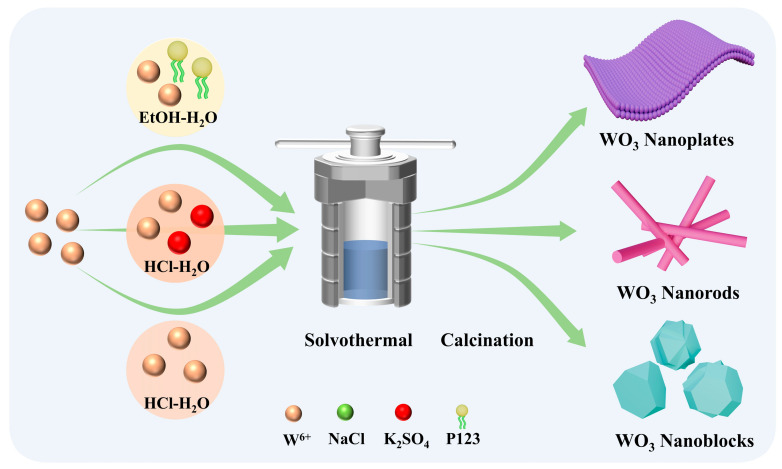
Schematic diagram of the synthesis of WO_3_ nanoplates, WO_3_ nanorods, and WO_3_ nanoblocks.

WO_3_ sensing materials with different morphologies were prepared by the solvothermal method (Figure 1): P123 micelles self-assemble into lamellar structures in the solvent, acting as structure-directing agents to guide the oriented two-dimensional growth of WO_3_ [32]. For WO_3_ nanorods, NaCl was employed as a structure-directing agent to induce and regulate nanocrystal growth, resulting in uniformly sized and structurally stable nanorods [33]. Furthermore, WO_3_ nanoblocks were synthesized without adding any additive during the hydrothermal reaction [34,35].

**Figure 2:**
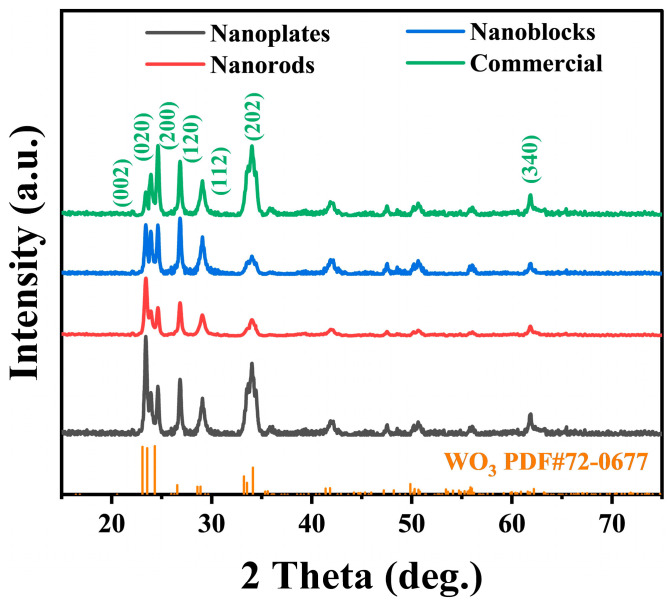
XRD patterns of WO_3_ nanoplates, WO_3_ nanorods, WO_3_ nanoblocks, and commercial WO_3_.

An XRD analysis was performed on three WO_3_ nanostructures with distinct morphologies (nanoplates, nanorods, and nanoblocks) and commercial WO_3_ to evaluate their phase compositions. The sharp characteristic diffraction peaks indicate that all materials exhibit good crystallinity (Figure 2) [36]. Additionally, the diffraction peaks of all samples match well with the monoclinic phase of WO_3_ (JCPDS No. 72-0677), confirming the successful synthesis of pure WO_3_ nanomaterials. According to the Scherrer equation, the average crystallite size of WO_3_ nanoplates calculated from the XRD pattern is the smallest, approximately 15.7 nm (Table S1).

**Figure 3:**
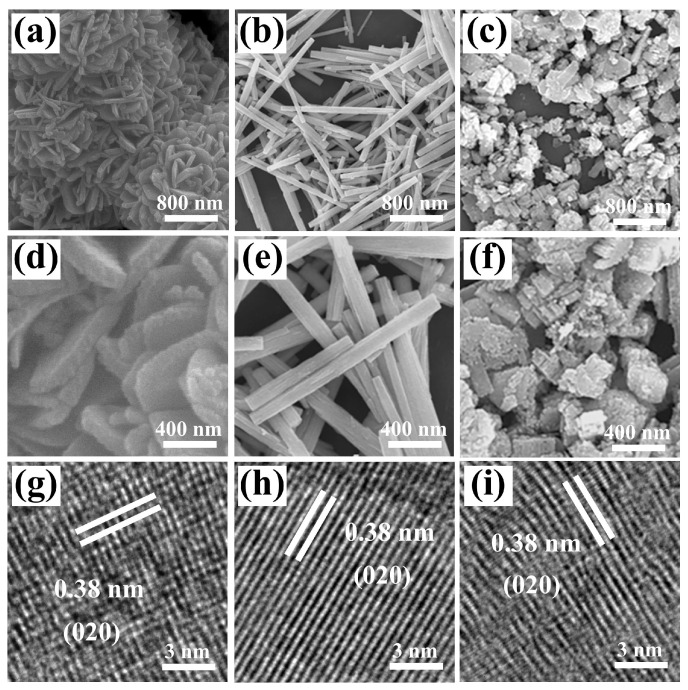
SEM images of (**a**,**d**) WO_3_ nanoplates, (**b**,**e**) WO_3_ nanorods, and (**c**,**f**) WO_3_ nanoblocks. HRTEM images of (**g**) WO_3_ nanoplates, (**h**) WO_3_ nanorods, and (**i**) WO_3_ nanoblocks.

SEM and TEM characterization provided further morphological images, enabling an in-depth analysis of their structural features. The images reveal that the nanoplates have a relatively uniform morphology, ranging in size from 600 to 800 nm (Figure 3a,d). Figure 3b,e show that the WO_3_ nanorods have diameters of 150–200 nm and lengths of 2–3 μm, exhibiting no significant agglomeration. In contrast, the WO_3_ nanoblocks exhibit an irregular and disordered morphology (Figure 3c,f). Moreover, as shown in Figure 3g–i, the lattice spacing of all three nanomaterials is 0.38 nm, matching the (020) crystal plane of the monoclinic WO_3_. This further reveals that the synthesized materials possess good crystallinity, consistent with the XRD results (Figure 2) [22].

**Figure 4:**
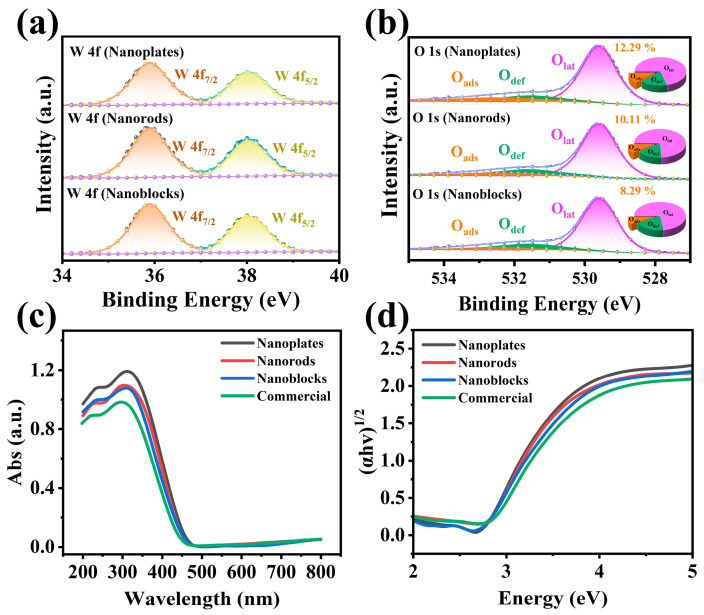
High resolution (**a**) W 4f and (**b**) O 1s XPS spectra of WO_3_ nanoplates, WO_3_ nanorods, and WO_3_ nanoblocks. (**c**) Solid-state UV-Vis diffuse reflectance spectroscopy response and (**d**) plots of (αhv)^1/2^ versus photon energy of WO_3_ nanoplates, WO_3_ nanorods, WO_3_ nanoblocks, and commercial WO_3_.

An XPS analysis was performed to investigate the synthesized sample’ surface chemical states. Figure S3 shows the complete spectrum, which reveals distinct signals corresponding to W 4f, W 4d, W 4p, O 1s, and C 1s, confirming the high purity of the prepared WO_3_. As shown in Figure 4a, the characteristic peaks of W 4f are present in the spectra of all three WO_3_ materials. The binding energies of W 4f are 35.91 eV and 37.90 eV for the nanoplates, 35.85 eV and 37.91 eV for the nanorods, 35.87 eV and 37.89 eV for the nanoblocks. The two characteristic peaks correspond to the W 4f_7/2_ and W 4f_5/2_ orbitals of W^6+^ in WO_3_, respectively [37,38]. The O 1s characteristic peaks of the three materials are shown in Figure 4b. After fitting, three binding energy positions emerge. Specifically, the binding energies for the nanoplates are 529.59 eV, 531.49 eV, and 532.39 eV; for the nanorods, they are 529.58 eV, 531.56 eV, and 532.65 eV; and for the nanoblocks, they are 529.58 eV, 530.56 eV, and 532.43 eV. These three distinct binding energy positions represent different chemical states of oxygen on the material surface, namely, lattice oxygen (O_lat_), defect oxygen (O_def_), and adsorbed oxygen (O_ads_) [39,40]. Lattice oxygen is typically stable and does not react with target gases. In contrast, adsorbed oxygen is more active on the surface of semiconductor materials and can interact with target gases, altering the concentration of surface charge carriers. Therefore, a higher concentration of adsorbed oxygen generally leads to better gas-sensing performance [22,41]. Based on the fitting results, the relative percentages of adsorbed oxygen in the three materials were calculated, as illustrated in the pie charts in Figure 4b. The nanoplates exhibited the highest proportion at 12.29%, followed by the nanorods (10.11%) and the nanoblocks (8.29%). Considering the differences in morphology and structure among these materials, the variation in adsorbed oxygen concentration may be related to their specific surface area [26].

Solid-state UV-Vis diffuse reflectance spectroscopy investigated the materials’ optical absorption characteristics and bandgap energy (Figure 4). The bandgap (*E_g_*) can be calculated using the following formula (Equation (1)) [26]:

(1) $${\left(\alpha \mathrm{hv}\right)}^{1/2}=A\left(\mathrm{hv}-E_{g}\right)$$

In the formula, α, h, ν, A, and E_g_ represent the absorption coefficient, Planck’s constant, light frequency, proportionality constant, and bandgap energy, respectively. The bandgap values of the four materials are presented in Figure 4d. The results demonstrate that commercial WO_3_ possesses the widest bandgap (2.81 eV), whereas the synthesized WO_3_ with different morphologies show slightly narrower bandgaps compared to commercial WO_3_, all clustering around 2.77 eV. A narrower bandgap facilitates the transition of charge carriers, thereby enhancing the gas-sensing performance [39]. This observation clearly indicates that morphology has negligible influence on the material’s bandgap during this synthesis process.

BET measurements were conducted on the four samples to investigate the effects of morphological control on the specific surface area and pore size of the materials (Figure S4). The specific surface areas of WO_3_ nanoplates, WO_3_ nanorods, WO_3_ nanoblocks, and commercial WO_3_ were measured to be 19.21 m^2^/g, 11.34 m^2^/g, 9.11 m^2^/g, and 6.35 m^2^/g, respectively. This demonstrates that morphological engineering significantly modulates the specific surface area, which regulates the population of surface-active sites, ultimately governing the gas-sensing performance [6,22]. Significantly, the N_2_ adsorption–desorption isotherm exhibits a Type IV curve with an H3 hysteresis loop, indicating the presence of a mesoporous structure in the WO_3_ nanoplates (Figure S4a) [39]. The mesoporous architecture results from the thermal elimination of the micelle-templated material formed framework by P123 [6,22,32].

**Figure 5:**
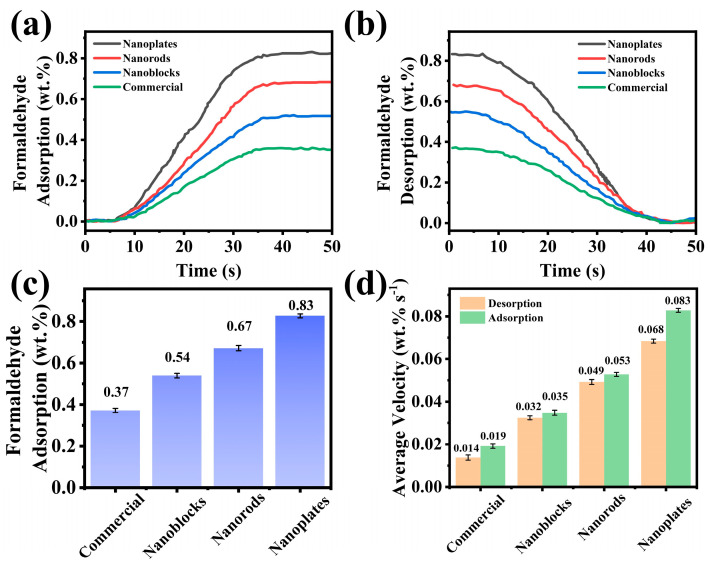
QCM adsorption–desorption tests of formaldehyde on WO_3_ nanoplates, WO_3_ nanorods, WO_3_ nanoblocks, and commercial WO_3_. (**a**) Adsorption curves, (**b**) desorption curves, (**c**) maximum adsorption capacity, and (**d**) adsorption/desorption rates.

QCM was further employed to investigate the effect of morphological variations in sensing materials on gas adsorption–desorption performance. Figure 5a–d display the formaldehyde adsorption–desorption curves of the four materials, along with their corresponding adsorption capacities and desorption rates. As shown in Figure 5c, the WO_3_ nanoplates exhibited the highest formaldehyde adsorption capacity at 0.83 wt%, followed by the nanorods (0.67 wt%) and nanoblocks (0.54 wt%), while the commercial WO_3_ demonstrated the lowest adsorption capacity (0.37 wt%). Furthermore, Figure 5d reveals that the WO_3_ nanoplates presented the fastest average adsorption–desorption rates (adsorption: 0.083 wt% s^−1^, desorption: 0.068 wt% s^−1^), whereas the commercial WO_3_ showed the slowest rates. These findings exhibited high consistency with the BET results (Figure S4). The larger surface area provides more active adsorption sites, consequently enhancing the adsorption capacity. Additionally, the abundant mesopores in WO_3_ nanoplates facilitate faster gas diffusion, enabling formaldehyde molecules to reach adsorption sites more rapidly [26,39,42].

### 3.2. Sensing Performance

Gas sensors were fabricated based on the synthesized WO_3_ nanomaterials with different morphologies (nanoplates, nanoblocks, and nanorods) and commercial WO_3_ to investigate their formaldehyde-sensing properties. The sensor performance was comprehensively evaluated by analyzing the optimal working temperature, selectivity, sensitivity, response/recovery time, long-term stability, and repeatability (see more details in the Supplementary Materials).

**Figure 6:**
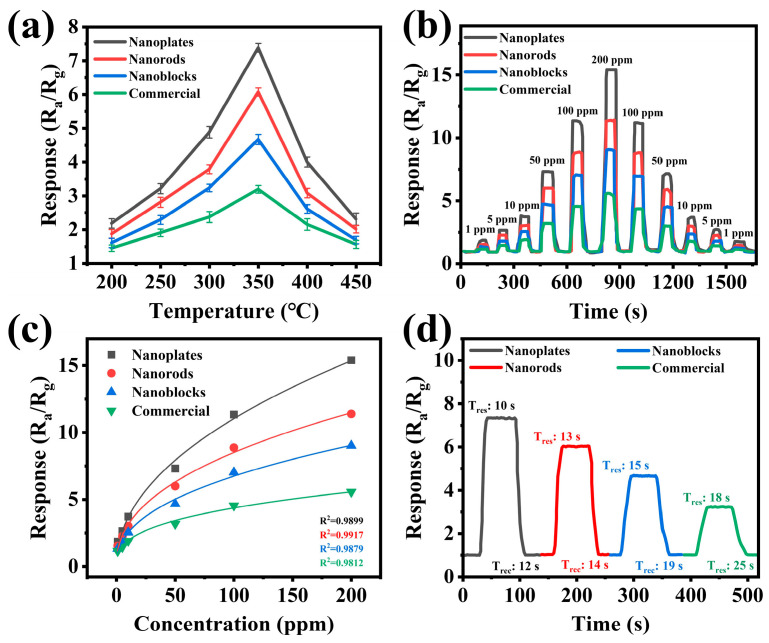
The sensing performance of the gas sensors based on WO_3_ nanoplates, WO_3_ nanorods, WO_3_ nanoblocks, and commercial WO_3_ for formaldehyde. (**a**) The response of the gas sensor to 50 ppm formaldehyde at different operating temperatures (200–450 °C). (**b**) The dynamic response curves of the sensor to different concentrations of formaldehyde (1–200 ppm) at 350 °C. (**c**) The relationship between the response of the gas sensor and formaldehyde concentration at 350 °C. (**d**) The sensor’s response and recovery time curve to 50 ppm formaldehyde at 350 °C.

The working temperature plays a decisive role in the performance of metal oxide semiconductor gas sensors [43]. The reaction rate is too slow at excessively low temperatures, while excessively high temperatures may cause material deactivation [42,44]. Figure 6a revealed the gas sensing performance of WO_3_-based sensors (nanoplates, nanorods, nanoblocks, and commercial) toward 50 ppm formaldehyde at different working temperatures (200–450 °C). The results demonstrated that the response values of all WO_3_ sensors initially increased and subsequently decreased with rising temperature, reaching a peak at 350 °C. Consequently, 350 °C was selected as the optimal working temperature for all subsequent gas-sensing tests. Furthermore, the WO_3_ nanoplate-based gas sensor exhibited the highest response (*R_a_*/*R_g_* = 7.4) toward 50 ppm formaldehyde, while the commercial WO_3_ showed the lowest response. The dynamic response curves of all four sensors to formaldehyde (1–200 ppm) were measured at the optimal operating temperature of 350 °C. As shown in Figure 6b, despite variations in sensitivity across different sensors, all demonstrated a concentration-dependent response to formaldehyde. In addition, when exposed to the same formaldehyde concentration in dynamic cycles, the sensors exhibited consistent response values, confirming their excellent reversibility and repeatability [26]. Notably, the WO_3_ nanoplate sensor retained a significant response value even at 500 ppb of formaldehyde (Figure S5).

In practical applications, the quantitative relationship between the target gas concentration and sensor response and the response/recovery characteristics are crucial performance metrics [45]. Figure 6c shows a strong correlation between the sensor response and formaldehyde concentration, validating its practical applicability for formaldehyde quantification (the fitting equation is shown in Table S2) [46]. In addition, Figure 6d shows the response/recovery times of the four sensors to 50 ppm formaldehyde. The results indicated that the gas sensor based on WO_3_ nanoplates exhibited the fastest response and recovery times (10 s/12 s), while the response/recovery time of the gas sensor based on commercial WO_3_ was the slowest (18 s/25 s). This enhancement can be attributed to the fact that two-dimensional nanoplates possess the largest specific surface areas (especially the transversely extended base surface), allowing gas molecules to be rapidly adsorbed across the entire surface with a very short diffusion path (without penetrating the material’s interior) [23,47]. At the same time, the two-dimensional mesoporous structure can provide a continuous conductive channel, which can quickly respond to the change in conductivity caused by surface gas reaction [23,47,48].

**Figure 7:**
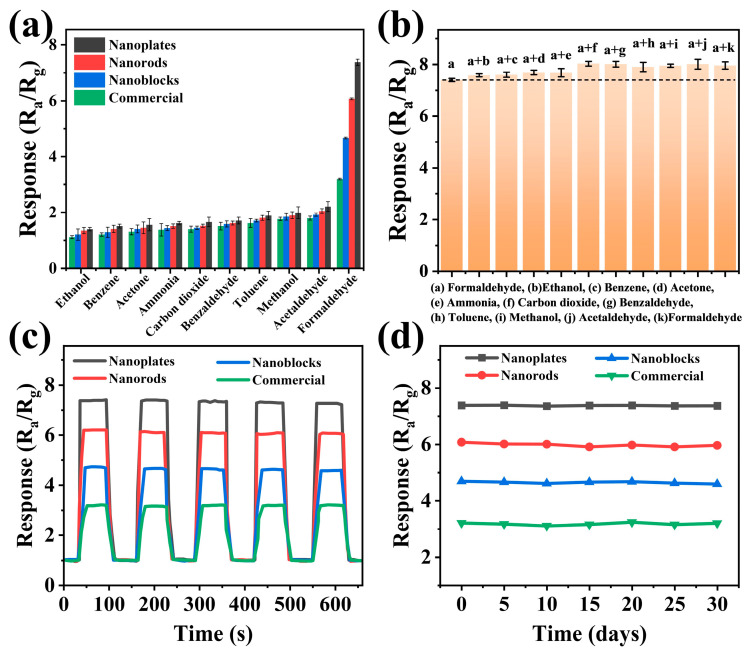
Gas-sensing properties of WO_3_ nanoplates, WO_3_ nanorods, WO_3_ nanoblocks, and commercial WO_3_ sensors. (**a**) Selectivity for 50 ppm formaldehyde and other interfering gases. (**b**) Identification test for the response to mixed gases containing 50 ppm of formaldehyde and 50 ppm of other interfering gases. (**c**) Reproducibility and (**d**) long-term stability for sensing 50 ppm formaldehyde. All tests were conducted at 350 °C.

To comprehensively evaluate the sensor performance, both selectivity tests and anti-interference tests were conducted, as these capabilities are critical for practical applications [49]. Figure 7a compares the responses of all sensors to 50 ppm ethanol, benzene, acetone, ammonia, carbon dioxide, benzaldehyde, toluene, methanol, acetaldehyde, and formaldehyde at 350 °C. Notably, all sensors demonstrated significantly higher responses to formaldehyde than to other test gases, with the WO_3_ nanoplate-based sensor exhibiting particularly outstanding selectivity. The anti-interference capability was further investigated (Figure 7b). The results revealed that the WO_3_ nanoplates sensor maintained remarkable stability, with response variations below 10% even when formaldehyde was mixed with various interfering gases. This highlights the excellent anti-interference ability of the WO_3_ nanoplate sensors and has broad application prospects [50]. Figure 7c presents five consecutive repeatability tests of gas sensors based on WO_3_ with different morphologies toward 50 ppm formaldehyde at 350 °C. It can be observed that the WO_3_ nanoplate-based sensor exhibited a minimal variation in the response amplitude, with stable response/recovery curves, demonstrating excellent repeatability. Figure 7d presents the sensitivity trends of the four sensors during periodic testing over one month under the same conditions (50 ppm formaldehyde, 350 °C). Notably, all sensors maintained robust long-term stability, particularly the WO_3_ nanoplate-based sensor, which showed the smallest response fluctuation. This outstanding repeatability and stability highlight the practical potential of WO_3_ nanomaterials in formaldehyde detection [51]. Furthermore, the response of the WO_3_ nanoplate sensor to 50 ppm formaldehyde was tested under different humidity levels (40–80%), as shown in Figure S6. The sensitivity of the WO_3_ nanoplate sensor decreased with increasing humidity, but the deviation remained below 6%, indicating the excellent humidity resistance of the sensor. Finally, Table S3 presents a comparison of the sensing performance of the WO_3_ nanoplates-based sensor developed in this study with previously reported MOS sensors for formaldehyde detection. As shown in the table, the WO_3_ nanoplate-based sensor exhibited higher sensitivity and a lower limit of detection for formaldehyde, demonstrating significant potential for practical applications compared to the formaldehyde-sensing characteristics reported in the earlier literature.

### 3.3. Practical Application

**Figure 8:**
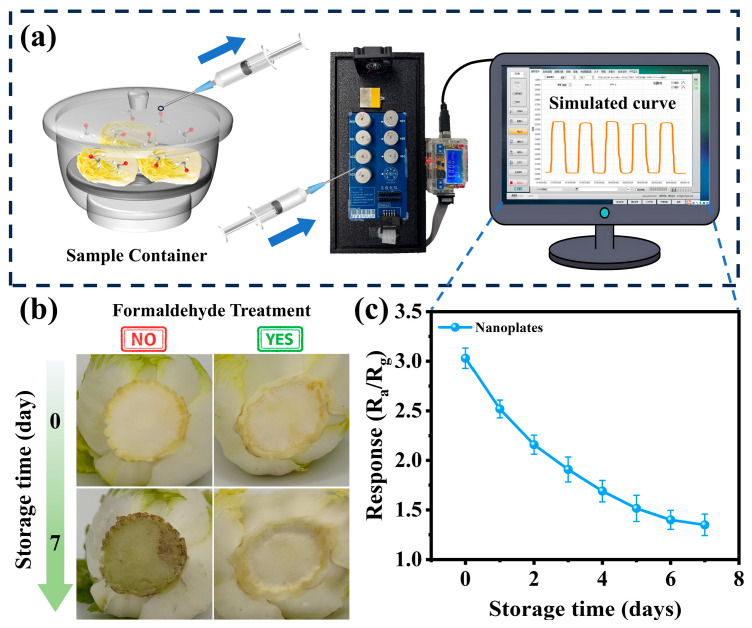
(**a**) Schematic diagram of the actual sample detection system. (**b**) Recording the changes in baby cabbage sprayed with formaldehyde and unsprayed at 0 and 7 days. (**c**) Curve showing the change in response for baby cabbage within 7 days after formaldehyde spraying.

To investigate the application of the sensor in practical detection, a MEMS sensor based on WO_3_ nanoplates was used to measure the formaldehyde concentration in baby cabbage contaminated with formaldehyde. Fresh market-sourced baby cabbage samples were treated with formaldehyde under laboratory conditions to replicate the illegal adulteration methods used by unscrupulous vendors. After removing the decayed leaves and roots from the purchased baby cabbage, the prepared formaldehyde solution (10 ppm) was sprayed on both the roots and outer surfaces before the samples were wrapped in plastic wrap and stored in cold storage (4 °C) [6,52]. The WO_3_ nanoplate sensor was used to conduct daily tests for seven consecutive days, with the data recorded once daily (Figure 8a). Due to its volatility, the actual formaldehyde concentration after addition was slightly lower than the nominal 10 ppm standard solution, as shown in the Day 0 data in Figure 8c. The decay rate of baby cabbage treated with the formaldehyde solution was significantly slower than that of the untreated control (Figure 8b). In Figure 8c, the sensor response gradually decreased due to formaldehyde volatilization [6,52]. International standards classify formaldehyde as a prohibited food additive, but adopt the ‘As Low As Reasonably Achievable’ principle for naturally occurring formaldehyde in certain foods (e.g., cod and tuna) or processing byproducts [53,54]. Therefore, the accurate detection of low-concentration formaldehyde is critical for ensuring food safety. WO_3_ nanoplate sensors exhibit high sensitivity and a low limit of detection (1.34@500 ppb), demonstrating significant practical potential for rapid formaldehyde detection in food.

### 3.4. Sensing Mechanism

**Figure 9:**
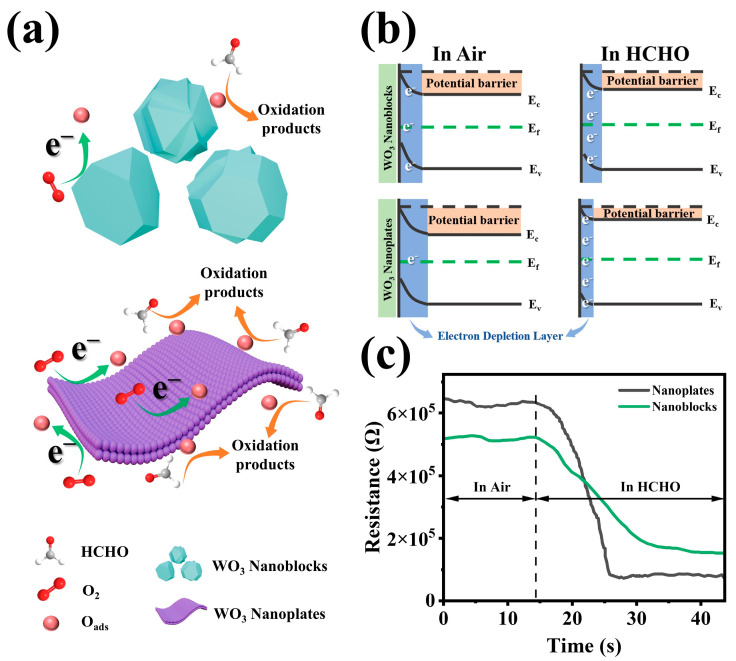
(**a**) Schematic illustration of the formaldehyde gas-sensing mechanism for WO_3_ nanoplates and WO_3_ nanoblocks. (**b**) Electronic structure evolution of WO_3_ nanoplates and WO_3_ nanoblocks in air and formaldehyde atmospheres. (**c**) Resistance response curves of WO_3_ nanoplates and WO_3_ nanoblocks after formaldehyde exposure.

WO_3_ is a typical n-type semiconductor, where electrical conduction is primarily governed by electrons in the conduction band [55]. The gas-sensing mechanism of WO_3_-based sensors is governed by the surface-controlled model [6,56,57]. Figure 9a illustrates the formaldehyde detection process mediated by surface reactions between the sensing material and gas molecules. When a WO_3_-based sensor is exposed to air, oxygen molecules adsorb onto the material’s surface. Given that the electron affinity of oxygen (A) exceeds the work function of WO_3_ (Φ), oxygen molecules extract electrons from the conduction band, forming various chemisorbed oxygen species (Equations (1)–(4)) [58]. This process induces upward band bending and generates an electron depletion layer (EDL) at the semiconductor surface, leading to a high-resistance state (Figure 9b) [22].

(2) $${O_{2}}_{\mathrm{gas}}\to {O_{2}}_{\mathrm{ads}}$$

(3) $${O_{2}}_{\mathrm{ads}}+e^{-}\to {O_{2}^{-}}_{\mathrm{ads}}$$

(4) $${O_{2}^{-}}_{\mathrm{ads}}+e^{-}\to {{2O}^{-}}_{\mathrm{ads}}$$

(5) $${O^{-}}_{\mathrm{ads}}+e^{-}\to {O^{2-}}_{\mathrm{ads}}$$

When exposed to the reducing gas formaldehyde, the chemisorbed oxygen species react vigorously with formaldehyde molecules. The formaldehyde is first oxidized to formic acid, before ultimately decomposing into CO_2_ and H_2_O (Equations (5) and (6)) [6,59].

(6) $${\mathrm{HCHO}}_{\mathrm{gas}}+{O^{-}}_{\mathrm{ads}}\to {\mathrm{CHOOH}}_{\mathrm{gas}}+e^{-}$$

(7) $${\mathrm{CHOOH}}_{\mathrm{gas}}+{O^{-}}_{\mathrm{ads}}\to {{\mathrm{CO}}_{2}}_{\mathrm{gas}}+H_{2}O_{\mathrm{gas}}+e^{-}$$

Throughout this reaction process, the released electrons return to the conduction band, decreasing the EDL thickness and reducing the sensor’s resistance (Figure 9b) [6]. Due to the thermal elimination of P123-templated micellar nanochannels, the WO_3_ nanoplates possess a unique mesoporous structure [6,22,32]. This unique mesoporous nanoplate structure provides a larger specific surface area (19.21 m^2^/g), thereby exposing more active sites [26]. As a result, more electrons in the conduction band are captured by oxygen and converted into chemically adsorbed oxygen, forming a thicker electron depletion layer, ultimately increasing baseline resistance [26,39]. Correspondingly, when the sensor is exposed to formaldehyde, the WO_3_ nanoplates can adsorb more formaldehyde molecules and undergo reactions. Consequently, more electrons are released into the conduction band, significantly reducing the EDL. Figure 4b, Figure 5, Figure 9c and Figure S4 provided favorable support for the above mechanistic analysis.

## 4. Conclusions

In summary, this study synthesized WO_3_ sensing materials with different morphologies and systematically investigated their formaldehyde gas-sensing performance. The materials were characterized by XRD, BET, TEM, and XPS to analyze their crystal structure, specific surface area, morphology, and chemical composition. Gas-sensing tests revealed that the WO_3_ nanoplate-based sensor exhibited high sensitivity (16.5@200 ppm), rapid response/recovery times (10 s/12 s), excellent selectivity, and a low limit of detection (500 ppb). The superior performance is primarily attributed to the unique morphological structure and mesoporous characteristics, which facilitate efficient gas adsorption/desorption, while the large specific surface area provides abundant active sites, which significantly amplify the sensor’s resistance variation. Furthermore, the sensor demonstrated a reliable formaldehyde detection capability in vegetable samples (baby cabbage), highlighting its potential for practical applications. Thus, this study provides a morphology-driven strategy for designing WO_3_-based sensors to detect formaldehyde in agricultural products, paving the way for advanced food safety and quality assessment technologies.

## Data Availability

Data are contained within the article and Supplementary Materials.

## Supplementary Materials

The following supporting information can be downloaded at https://www.mdpi.com/article/10.3390/bios15070400/s1, the “Synthesis of WO_3_ Nanorods”, “Synthesis of WO_3_ Nanoblocks”, and “Gas sensor measurement” sections; Figure S1. (a) The measuring circuit of the MEMS gas sensor. (b) Panoramic view of the MEMS gas sensor test system. (c) Exploded views of the MEMS gas sensor. Figure S2. Actual photos of the testing experimental setup. Figure S3. XPS full spectra of WO_3_ nanoplates, WO_3_ nanorods, and WO_3_ nanoblocks. Figure S4. N_2_ adsorption/desorption isotherms of (a) WO_3_ nanoplates, (b) WO_3_ nanorods, (c) WO_3_ nanoblocks, and (d) commercial WO_3_. The corresponding illustrations show the respective pore sizes. Figure S5. The limit of detection of WO_3_ nanoplates toward 500 ppb formaldehyde at 350 °C. Figure S6. The response of WO_3_ nanoplate-based sensors to 50 ppm of HCHO at different humidity levels. Table S1. The average crystallite sizes of WO_3_ nanoplates, WO_3_ nanorods, WO_3_ nanoblocks, and commercial WO_3_. Table S2. The fitting equations for the relationship between the response of gas sensors and formaldehyde concentration. Table S3. Comparison of the gas-sensing performance of MOS-based sensors to formaldehyde. References [60,61,62,63,64,65,66,67] are cited in the Supplementary Materials.
