# Supplementary material for: Controlled Synthesis of Tungsten Oxide Nanomaterials with Different Morphologies and Their Gas-Sensing Properties for Formaldehyde in Vegetables
Source: Biosensors (Basel). 2025 Jun 20;15(7):400. doi: 10.3390/bios15070400 (PMC12293331; doi:10.3390/bios15070400)
Supplement: Supplementary file 1 [file biosensors-15-00400-s001.zip › biosensors-3664262-supplementary.pdf]

Supplementary Material

Synthesis of WO_3 nanorods

The WO_3 nanorods were synthesized as follows: First, 0.825 g of $Na_2WO_4·2H_2O$ and 0.29 g of NaCl were mixed in 19 mL of deionized water. Then, 3 mol/L hydrochloric acid (HCl) was added dropwise to the mixture until the pH reached 2.0. The solution was transferred into a 50 mL autoclave and heated at 180 °C for 24 h. After the reaction, the product was collected, washed, and calcined (400 °C for 2 h, the heating rate is 5 °C /min) to obtain the WO_3 nanorods.

Synthesis of WO_3 nanoblocks

First, 0.005 mol of $Na_2WO_4·2H_2O$ was completely dissolved in 50 mL of deionized water. Then, 10 mL of a 3 M HCl solution was added dropwise to the mixture, followed by additional stirring for 30 min. The resulting solution was transferred into a Teflon-lined autoclave and maintained at 140 °C for 6 h. After the completion of the hydrothermal reaction, the precipitate was collected by centrifugation, thoroughly washed with deionized water and ethanol alternately, and dried at 60 °C. Finally, the obtained powder was calcined in a muffle furnace at 400 °C for 2 h (the heating rate was 5 °C /min).

Gas sensor measurement

The gas sensing performance of gas sensors was measured through a static testing system that can record real-time changes in sensor resistance. Different steam concentrations were obtained by injecting a liquid with a volume of Q into the testing chamber. Equation S1 was used to determine the volume Q:

$$\boldsymbol{Q} = \frac{V \times C \times M}{22.4 \times d \times \rho} \times \frac{273+T_R}{273+T_B} \times \mathbf{10^{-9}} \qquad \text{(S1)}$$

In the formula, V, C, M, d, ρ, T_R, and T_B are the volume of the test chamber (1 L), vapor concentration (ppm), liquid molecular weight, liquid density, liquid purity, ambient temperature, and test chamber temperature, respectively. For testing reducing gases with n-type semiconductor gas sensors, the sensor's response value is defined as $S=R_a/R_g$, where R_a is the sensor's resistance in air and R_g is its resistance in reducing gases. The response and recovery times are defined as the time it takes for a gas sensor to achieve resistance changes from R_a to R_a-90% (Ra-Rg) and from R_g to R_g+90% (R_a-R_g) when adsorbing and desorbing the target gas, respectively. The relationship between resistance (R) and voltage (V_{out}) is shown in Equation S2:

$$\boldsymbol{R} = \frac{(5-V_{out})}{V_{out}} \times \boldsymbol{R_L} \qquad \text{(S2)}$$

The limit of detection (LOD) of semiconductor gas sensors is generally defined as the minimum gas concentration that produces a detectable signal above the noise level, and is typically calculated using three times the signal-to-noise ratio. For the WO_3 nanoplate sensor, the response to low-concentration formaldehyde exhibits a linear relationship described by the equation y = 0.2067x + 1.6357 (R^2 = 0.9986), where y represents the sensor response and x denotes the formaldehyde concentration (ppm). With a calculated noise standard deviation of 0.0339, the limit of detection is determined as follows: LOD = 3 × (0.0339/0.2067) = 0.4920 ppm<500 ppb.

Figure captions:

Figure S1. (a) The measuring circuit of the MEMS gas sensor. **(b)** Panoramic view of the MEMS gas sensor test system. **(c)** Exploded views of the MEMS gas sensor.

Figure S2. Actual photos of the testing experimental setup.

Figure S3. XPS full spectra of WO_3 nanoplates, WO_3 nanorods, and WO_3 nanoblocks.

Figure S4. N_2 adsorption/desorption isotherms of **(a)** WO_3 nanoplates, **(b)** WO_3 nanorods, **(c)** WO_3 nanoblocks, and **(d)** commercial WO_3. The corresponding illustrations show the respective pore sizes.

Figure S5. The limit of detection of WO_3 nanoplates toward 500 ppb formaldehyde at 350 °C.

Figure S6. The response of WO_3 nanoplate-based sensors to 50 ppm of HCHO at different humidity levels.

Table captions:

Table S1. The average crystallite sizes of WO_3 nanoplates, WO_3 nanorods, WO_3 nanoblocks, and commercial WO_3.

Table S2. The fitting equations for the relationship between the response of gas sensors and formaldehyde concentration.

Table S3. Comparison of the gas-sensing performance of MOS-based sensors to formaldehyde.

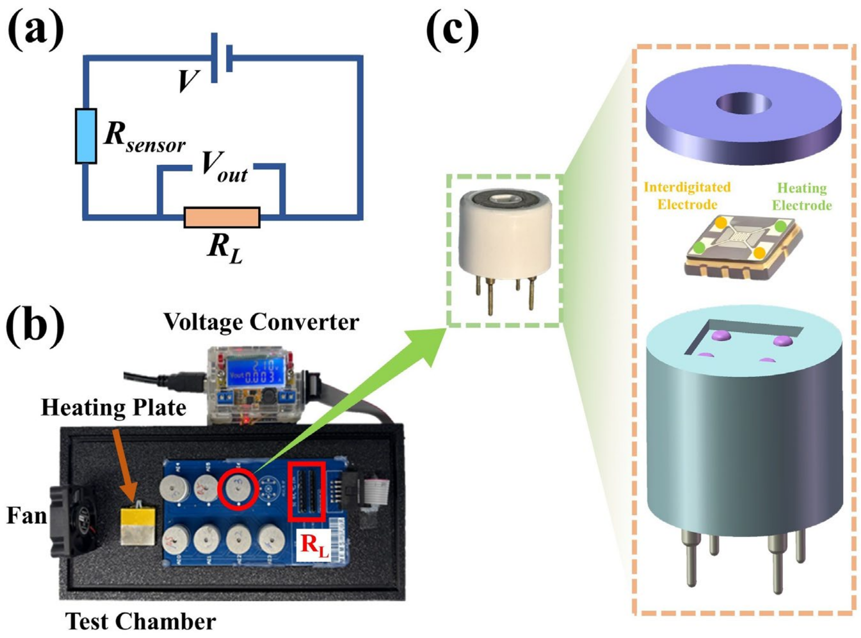

Figure S1. **(a)** The measuring circuit of the MEMS gas sensor. **(b)** Panoramic view of the MEMS gas sensor test system. **(c)** Exploded views of the MEMS gas sensor.

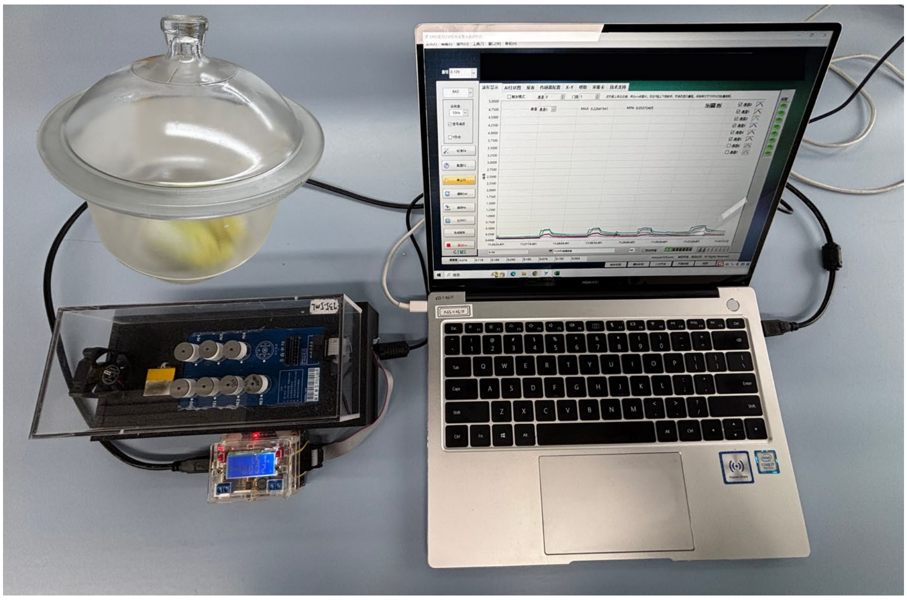

Figure S2. Actual photos of the testing experimental setup.

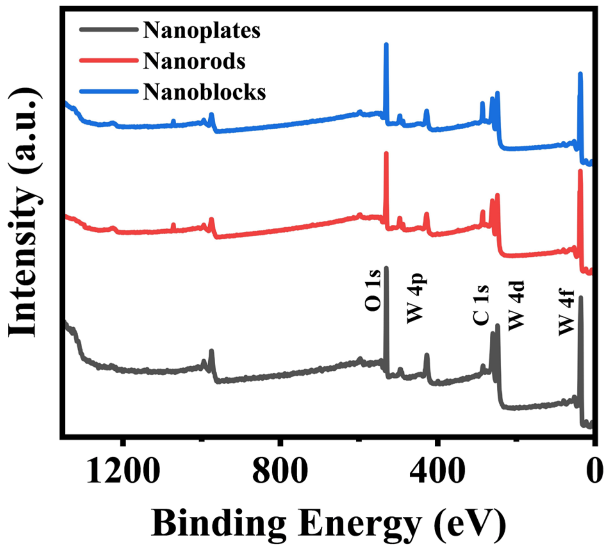

Figure S3. XPS full spectra of WO_3 nanoplates, WO_3 nanorods, and WO_3 nanoblocks.

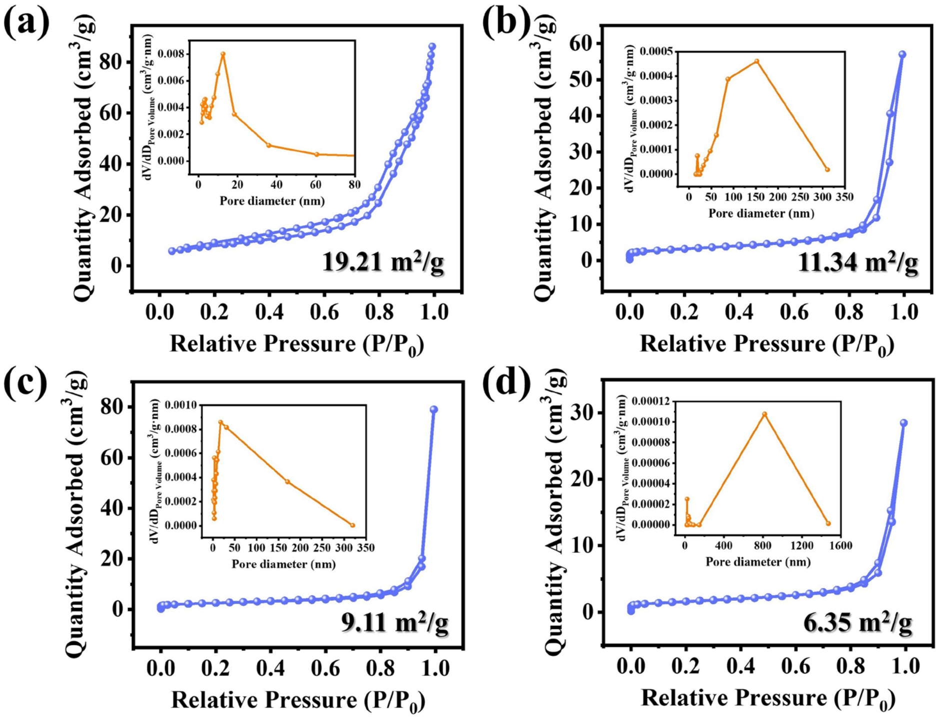

Figure S4. N_2 adsorption/desorption isotherms of **(a)** WO_3 nanoplates, **(b)** WO_3 nanorods, **(c)** WO_3 nanoblocks, and **(d)** commercial WO_3. The corresponding illustrations show the respective pore sizes.

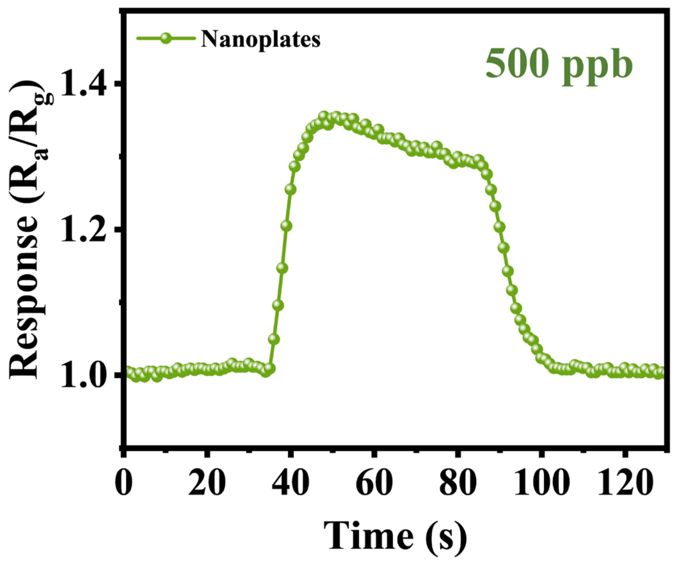

Figure S5. The limit of detection of WO_3 nanoplates toward 500 ppb formaldehyde at 350 °C.

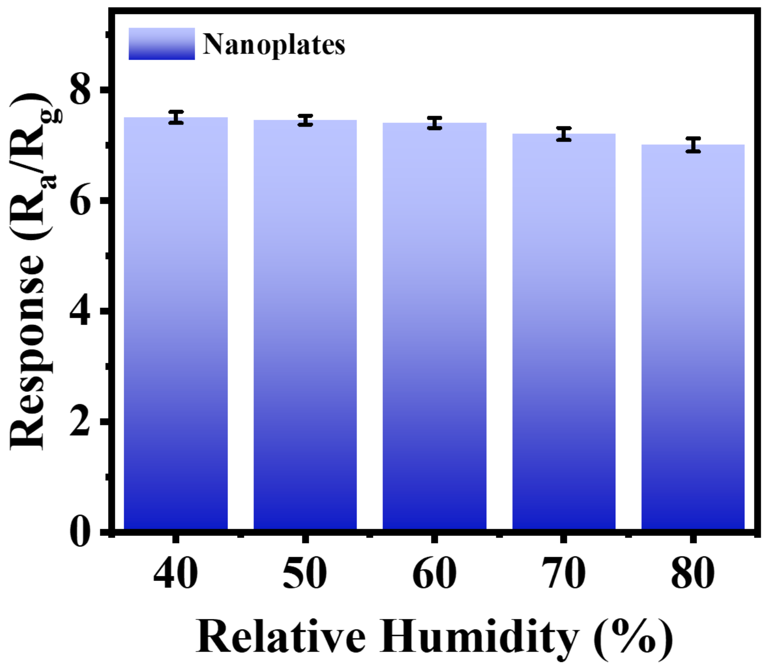

Figure S6. The response of WO_3 nanoplate-based sensors to 50 ppm of HCHO at different humidity levels.

Table S1. The average crystallite sizes of WO_3 nanoplates, WO_3 nanorods, WO_3 nanoblocks, and commercial WO_3.

**Materials**	**Average Crystal Size (nm)**
Nanoplates	15.7
Nanorods	16.9
Nanoblocks	18.9
Commercialized	19.2

Table S2. The fitting equations for the relationship between the response of gas sensors and formaldehyde concentration.

Materials	Fitting curve equation	R^2
Nanoplates	$y=\frac{3397.3143\times3.6062\times10^{-4}\times x^{1-0.5219}}{1+3.6062\times10^{-4}\times x^{1-0.5219}}$	0.9899
Nanorods	$y=\frac{941.8527\times0.0012\times x^{1-0.5643}}{1+0.0012\times x^{1-0.5643}}$	0.9917
Nanoblocks	$y=\frac{1395.9360\times6.8265\times10^{-4}\times x^{1-0.5740}}{1+6.8265\times10^{-4}\times x^{1-0.5740}}$	0.9879
Commercialized	$y=\frac{1092.8192\times7.9349\times10^{-4}\times x^{1-0.6479}}{1+7.9349\times10^{-4}\times x^{1-0.6479}}$	0.9812

Table S3. Comparison of the gas-sensing performance of MOS-based sensors to formaldehyde.

Materials	Temp. (°C)	Conc. (ppm)	Response (Ra/Rg)	Tres/Trec (s)	Detection limit (ppm)	Ref.
Co_3O_4 hollow nanocages	180	100	5	37/42	4	S1
WO_3 2D nanosheets	400	100	6.48	15/10	/	S2
In_2O_3 nanoribbons	340	100	3.13	18/17	/	S3
ZnO nanosheets	240	100	3	20/50	2	S4
SnO_2/Zn_2SnO_4 nanorods	162	100	8.1	35/78	1	S5
Ag-In_2O_3/ZnO nanoparticles	300	100	7.4	15/4	1	S6
Zn_2SnO_4 nanospheres	160	100	10	/	2	S7
In_2O_3 nanoparticles	400	100	5	/	1	S8
CuO/SnO_2 nanowires	250	50	2.42	52/80	1.5	S9
NiO flowers	200	100	3.5	30/56	/	S10
WO_3 nanoplates	350	50	7.4	10/12	0.5	This work

References

S1. Cao, J.; Zhang, N.; Wang, S.; Zhang, H. Electronic Structure-Dependent Formaldehyde Gas Sensing Performance of the In_2O_3/Co_3O_4 Core/Shell Hierarchical Heterostructure Sensors. *J. Colloid Interface Sci.* **2020**, *577*, 19–28, doi:10.1016/j.jcis.2020.05.028.

S2. Yu, H.; Li, J.; Li, Z.; Tian, Y.; Yang, Z. Enhanced Formaldehyde Sensing Performance Based on Ag@WO_3 2D Nanocomposite. *Powder Technol.* **2019**, *343*, 1–10, doi:10.1016/j.powtec.2018.11.008.

S3. Li, Z.; Fan, Y.; Zhan, J. In_2O_3 Nanofibers and Nanoribbons: Preparation by Electrospinning and Their Formaldehyde Gas-Sensing Properties. *Eur. J. Inorg. Chem.* **2010**, *2010*, 3348–3353, doi:10.1002/ejic.201000313.

S4. Chen, Z.-W.; Hong, Y.-Y.; Lin, Z.-D.; Liu, L.-M.; Zhang, X.-W. Enhanced Formaldehyde Gas Sensing Properties of ZnO Nanosheets Modified with Graphene. *Electron. Mater. Lett.* **2017**, *13*, 270–276, doi:10.1007/s13391-017-6245-z.

S5. Xiao, X.; Xing, X.; Han, B.; Deng, D.; Cai, X.; Wang, Y. Enhanced Formaldehyde Sensing Properties of SnO_2 Nanorods Coupled with Zn_2SnO_4. *RSC Adv.* **2015**, *5*, 42628–42636, doi:10.1039/C5RA01887K.

S6. Dong, C.; Liu, X.; Han, B.; Deng, S.; Xiao, X.; Wang, Y. Nonaqueous Synthesis of Ag-Functionalized In_2O_3/ZnO Nanocomposites for Highly Sensitive Formaldehyde Sensor. *Sens. Actuators, B* **2016**, *224*, 193–200, doi:10.1016/j.snb.2015.09.107.

S7. Li, X.; Zhang, N.; Liu, C.; Adimi, S.; Zhou, J.; Liu, D.; Ruan, S. Enhanced Gas Sensing Properties for Formaldehyde Based on ZnO/Zn_2SnO_4 Composites from One-Step Hydrothermal Synthesis. *J. Alloys Compd.* **2021**, *850*, 156606, doi:10.1016/j.jallcom.2020.156606.

S8. Gong, F.; Liu, H.; Liu, C.; Gong, Y.; Zhang, Y.; Meng, E.; Li, F. 3D Hierarchical In_2O_3Nanoarchitectures Consisting of Nanocuboids and Nanosheets for Chemical Sensors with Enhanced Performances. *Mater. Lett.* **2016**, *163*, 236–239, doi:10.1016/j.matlet.2015.10.106.

S9. Zhu, L.-Y.; Yuan, K.; Yang, J.-G.; Ma, H.-P.; Wang, T.; Ji, X.-M.; Feng, J.-J.; Devi, A.; Lu, H.-L. Fabrication of Heterostructured P-CuO/n-SnO_2 Core-Shell Nanowires for Enhanced Sensitive and Selective Formaldehyde Detection. *Sens. Actuators, B* **2019**, *290*, 233–241, doi:10.1016/j.snb.2019.03.092.

S10. San, X.; Zhao, G.; Wang, G.; Shen, Y.; Meng, D.; Zhang, Y.; Meng, F. Assembly of 3D Flower-like NiO Hierarchical Architectures by 2D Nanosheets: Synthesis and Their Sensing Properties to Formaldehyde. *RSC Adv.* **2017**, *7*, 3540–3549, doi:10.1039/C6RA25883B.
